# Categories or dimensions? A critical systematic review of taxometric evidence in autism, ADHD, and language disorders

**DOI:** 10.1002/jcv2.70177

**Published:** 2026-10-08

**Authors:** Enrico Toffalini, Margherita Calderan, Riccardo Pagan, Valentina Tobia, Edmund Sonuga‐Barke

**Affiliations:** ^1^ Department of General Psychology University of Padua Padua Italy; ^2^ Department of Social and Developmental Psychology University of Padua Padua Italy; ^3^ Faculty of Psychology University Vita‐Salute San Raffaele Milan Italy; ^4^ Institute of Psychiatry, Psychology & Neuroscience King's College London London UK

**Keywords:** ADHD, autism, language impairment, neurodevelopmental conditions, taxometric analysis

## Abstract

**Background:**

Taxometric analysis is designed to test whether the latent structure underlying clinical conditions is categorical (taxonic) or dimensional. Despite its relevance to ongoing debates on neurodevelopmental conditions, its application to this field remains limited.

**Methods:**

We conducted a preregistered systematic review (OSF) searching PsycINFO, PubMed, Scopus, and Web of Science from inception to April 2025, with a supplementary search made in January 2026. We included peer‐reviewed empirical studies applying at least one taxometric procedure (e.g., MAMBAC, MAXEIG, MAXCOV, L‐Mode) to neurodevelopmental conditions/traits and reporting a categorical versus dimensional conclusion (or sufficient indices to infer it). Two reviewers independently screened and extracted data. Study quality (risk of bias for taxometric inference) was appraised using an ad hoc checklist focused on key threats, especially artificial admixture/compound sampling, and on reporting of indicator validity, nuisance covariance, and skewness. Findings were synthesized descriptively.

**Results:**

We identified 126 records across the main database search (*n* = 110) and the supplementary search (*n* = 16). Fourteen studies were included. ADHD and language impairment studies largely supported dimensional models, whereas most autism and subtype studies reported taxonic patterns. Artificial admixture was very common in studies reaching taxonic conclusions, especially for autism. In Monte Carlo simulations, admixture consistently generated false‐positive taxonic findings from dimensional data.

**Conclusion:**

Based on the best available evidence, ADHD and language impairment appear continuous dimensions rather than discrete categories. Evidence for a categorical structure is stronger for autism, but a definitive conclusion is frustrated by sampling‐related biases, especially artificial admixture. More population‐based, methodologically transparent taxometric studies are needed.

## Key Points

**What's known?**

- Neurodevelopmental diagnostic categories have recently been questioned in favour of dimensional models. Taxometric analysis is explicitly designed to test whether disorders are categorical or dimensional in nature.

**What's new?**

- The authors identified 14 studies. ADHD and language impairment consistently showed dimensional findings. Autism more often showed taxonic patterns, but these were typically observed in diagnosis‐structured designs with high risk of artificial admixture, which we show can create false taxonic results.

**What's relevant?**

- The best available evidence supports dimensionality for ADHD and language impairment. Apparent categorical structure in autism is more frequently reported, but its interpretation is frustrated by sampling‐related biases, especially artificial admixture, with direct implications for how diagnostic boundaries are justified in research and practice.

## INTRODUCTION

In recent years, there has been a reconsideration of the conceptual foundations of neurodevelopmental conditions (NDCs). The notion that diagnostic entities defined in the DSM‐5 reflect discrete categories with identifiable non‐arbitrary ‘natural’ boundaries, separating disorder from non‐disorder, and one disorder from another (American Psychiatric Association, 2013) has increasingly been questioned in favor of dimensional and transdiagnostic perspectives (e.g., Astle et al., 2022; Caviola et al., 2024; Happé & Frith, 2020, 2021; Mammarella et al., 2021; Michelini et al., 2024; Posner et al., 2020; Sonuga‐Barke, 2020). In fact, the issue has been discussed for a long time. Sonuga‐Barke (1998) questioned whether a medical model that frames childhood conditions as categorical disorders intrinsically linked to dysfunction was appropriate and useful. While diagnostic manuals such as the DSM are presented as atheoretical, they in practice adopt a “neo‐Kraepelinian” vision of psychopathology, treating disorders as natural disease entities (Coghill & Sonuga‐Barke, 2012).

In a dimensional view, traits relevant to NDCs vary continuously in the population, and clinically diagnosed cases lie at the extreme end of these distributions. Consequently, diagnostic decisions depend on where thresholds are placed and on the operationalization of impairment, both of which are partly arbitrary and context dependent, even when clinical needs are real (Arildskov et al., 2022; Haslam et al., 2006). Consistent with changes in diagnostic recognition and ascertainment, autism diagnoses have increased over time, including among higher‐functioning individuals (Russell et al., 2022), indicating historical changes in ascertainment and the composition of diagnosed samples.

Given the broadly dimensional and overlapping liabilities supported by current neurodevelopmental and behavioral genetic research, the key question becomes whether any apparent discontinuities may still emerge to reflect genuine phenotypic breaks, or whether such discontinuities reflect artifacts. If underlying traits are continuous, discrete diagnoses may still remain relevant in practice (for access to services, educational accommodations, and clinical decision‐making), but they should not be interpreted as evidence of natural discontinuities (Caviola et al., 2024). A key goal, therefore, is to distinguish the practical value of diagnosis from claims about latent categorical structure.

Several lines of evidence support a dimensional and overlapping view of NDCs. Individuals sharing the same diagnostic label often show substantial heterogeneity in symptom profiles, cognitive functioning, and developmental trajectories, and diagnostic boundaries are mostly blurred by comorbidity (e.g., Astle et al., 2022; Coghill & Sonuga‐Barke, 2012; Michelini et al., 2024). This picture is consistent with multifactorial and polygenic models of developmental psychopathology, that highlight extreme pleiotropy and genetic overlap across neurodevelopmental traits (Astle & Fletcher‐Watson, 2020; Caviola et al., 2024; Cross‐Disorder Group of the Psychiatric Genomics Consortium, 2019; Demontis et al., 2023; Kovas & Plomin, 2006; Pennington et al., 2012; Plomin & Kovas, 2005). This polygenic‐pleiotropic picture is very straightforward for ADHD; this also holds for Autism Spectrum Disorder (ASD), which however may additionally include a restricted subset of cases carrying rare high‐impact variants (yet distributed among many different loci, and generally accompanied by broader neurodevelopmental impairment; Satterstrom et al., 2020), which could lead to some taxonic‐looking patterns in clinical samples.

Despite very weak overall support for categorical etiologies, categorical thinking still prevails in research practices (case‐control comparisons) and dominates parts of the neurodevelopmental literature, including debates about ASD, proposed subtypes within NDCs, and emerging data‐driven clusters (Astle et al., 2022; Burgess et al., 2018; Chown & Leatherland, 2021; Frazier et al., 2023; e.g., Happé & Frith, 2021). The same question applies across NDCs: learning and language disorders, intellectual disability, and tic severity are routinely assessed using continuous psychometric measures (Caviola et al., 2024; Mammarella et al., 2021), yet the (non‐)arbitrariness of their diagnostic thresholds has rarely been tested directly: debates on dyslexia subtypes and on cut‐offs for language impairment parallel the autism debate (Burgess et al., 2018; Dollaghan, 2004). Transdiagnostic models place all these conditions on a common neurodevelopmental spectrum (Michelini et al., 2024). These developments align neurodevelopmental research with broader transdiagnostic dimensional frameworks (e.g., RDoC, HiTOP), which describe psychopathology as continua spanning typical variation to impairment (Insel et al., 2010; Kotov et al., 2017).

Taxometric methods were developed to test whether observed clinical indicators are more consistent with a latent category (taxon) or with a continuous dimension (Meehl, 1995). Rather than assuming groups a priori, taxometrics evaluates whether multivariate relationships among indicators show discontinuities that would be expected if a latent boundary separated two qualitatively distinct groups, or instead vary smoothly across the full range of severity (Ruscio et al., 2018). Well known taxometric methods include MAMBAC (Mean Above Minus Below A Cut), MAXCOV (Maximum Covariance), MAXEIG (Maximum Eigenvalue), and L‐Mode (Latent Mode Factor Analysis) (Cole, 2004; Ruscio, 2007; Ruscio & Ruscio, 2004a); recently, the simulation‐based Comparison Curve Fit Index (CCFI) has been introduced to provide quantitative indices for inference (Ruscio et al., 2018).

In adult psychopathology, taxometric studies have generally supported dimensional structures for most conditions (Haslam et al., 2012, 2020). In neurodevelopmental research, however, taxometrics has been used rarely, despite repeated calls to use explicit tests when debating categorical versus dimensional interpretations of childhood conditions (Beauchaine, 2003; Coghill & Sonuga‐Barke, 2012; Sonuga‐Barke, 1998). In their famous debate on whether ASD reflects a category or a continuum, Happé and Frith (2021) and Chown and Leatherland (2021) never mention taxometry. Astle et al. (2022) briefly acknowledge it, but predominantly emphasize studies using cluster analysis (i.e., data‐driven procedures aimed at grouping individuals based on multivariate simularity). The limitations of clustering methods for assessing taxonicity, however, have been discussed for decades: clustering algorithms impose (rather than test for) categorical structures, they provide weak tests of taxonicity, and fail to recover known taxa in simulations (Beauchaine, 2003; Ruscio & Ruscio, 2004b). Simulations using psychometrically realistic distributions suggest that common clustering methods inflate the number of detected clusters (Toffalini et al., 2022, 2024).

Like any inferential approach, taxometric conclusions are sensitive to design and measurement features. Sources of bias include poor indicator validity, nuisance covariance, low base rate, and artificial admixture/compound sampling (Cole, 2004; Haslam et al., 2020; Ruscio et al., 2018; Ruscio & Ruscio, 2004a). Poor indicator validity implies that indicators present little mean difference between individuals with and without the target condition. Nuisance covariance indicates strong residual correlations among indicators after accounting for group membership, indicating redundancy. Low base rate implies that too few individuals in the sample belong to the taxon; what matters is their absolute number rather than their proportion, so large samples partly compensate (Meehl & Yonce, 1994). Finally, admixture/compound sampling indicate that participants are combined from different recruitment sources or selection criteria (often as an attempt to compensate for a low base rate). Ruscio and Ruscio (2004a) list three such sampling practices that lead to pseudotaxonic results: when clinical and nonclinical participants are separately recruited and merged into a single sample; when a sample is split into subsamples based on different selection criteria and these are then used to test taxometric questions (“subtractive” compound sampling; Haslam et al., 2020); and when researchers remove observations from the putative complement (“control participants”) to inflate the base rate of the taxon. All these scenarios artificially impose categorical structures onto data that might be strictly dimensional.

### Goals of the present review

We aimed to critically review the use of taxometric methods in published studies of NDCs. The preregistered search focused on the most commonly investigated categories: Autism Spectrum Disorder (ASD), Attention‐Deficit/Hyperactivity Disorder (ADHD), and Learning Disorders, and was subsequently broadened through a targeted supplementary search covering Intellectual Disability, Tourette/Tic Disorders, and Language Impairment/Disorder. Eligible evidence permitted substantive synthesis mainly for ADHD, autism, and language impairment, while evidence for other NDCs was absent or very limited, a scarcity that itself reveals an important gap in the literature.

A crucial goal was to assess the methodological quality and credibility of taxometric findings. We evaluated potential sources of bias, such as artificial admixture, and assessed whether studies provided methodological information to evaluate other known threats to validity: low indicator validity, high nuisance covariance, indicators skewness (extreme skewness can also create the illusion of categorical structures, Ruscio & Ruscio, 2004a, 2004b; this might be relevant when assessing rare or extreme behaviors).

Finally, we conducted a Monte Carlo simulation study to empirically illustrate the extent to which artificial admixture can lead to the risk of reaching taxonic conclusions in otherwise dimensional data. We simulated a dimensional scenario where clinical and nonclinical participants are separately recruited and merged into a single sample; taxometric (and model‐based cluster) analyses were then performed on a set of observed indicators reflecting the latent clinical dimension.

## METHODS

The systematic review was conducted in accordance with the *Preferred Reporting Items for Systematic Reviews and Meta‐Analyses* (PRISMA) 2020 guidelines. A review protocol was preregistered on OSF registries at: https://osf.io/65y9g/?view_only=91e97c6b00dc444abb83fefcf67cbac0.

### Sources and search strategy

A systematic search for relevant peer‐reviewed articles was conducted on April 14^th^, 2025 using four electronic databases: PsycINFO, PubMed, Scopus, and Web of Science. No time limits were applied. The following search query, reflecting the methods and populations of interest, was applied to titles, abstracts, keywords:

((“taxometric*” OR “MAMBAC” OR “MAXCOV” OR “MAXEIG” OR “L‐Mode”) AND (“autis*” OR “ASD” OR “attention‐deficit*” OR “ADHD” OR “learning dis*” OR “reading dis*” OR “dyslexia” OR “math* dis*” OR “math* diff*”))

In PubMed, where the wildcard * was not supported, it was replaced with full terms (e.g., “disorder”, “disability” for “dis*”; “mathematical”, “mathematics”, “math” for “math*”).

Additionally, as a deviation from the preregistered protocol, a targeted supplementary search (January 20th, 2026) was conducted using the same taxometric terms and replacing the condition‐specific component of the query with:

(“language impairment” OR “language disorder*” OR “specific language impairment” OR “intellectual disability” OR “intellectual developmental disorder” OR “developmental delay” OR “general cognitive impairment” OR “Tourette*” OR “tic disorder*” OR “chronic tic*” OR “motor tic*” OR “vocal tic*”)

Additionally, the full search query was launched on Google Scholar, and the first 10 pages of results were screened manually. References cited in, and articles citing, Haslam et al. (2020) were also screened via Google Scholar. Finally, an exploratory search using OpenAI's GPT‐4o “Deep Research” tool was conducted, providing the preregistration protocol as context. No additional articles of interest were found through these means.

### Eligibility criteria

Inclusion criteria were.

1. The study applied at least one taxometric procedure (MAMBAC, MAXCOV, MAXEIG, L‐Mode, MAXSLOPE; other procedures would be considered if explicitly described as taxometric);
2. The target population consisted of individuals with NDCs/traits;
3. The study drew a conclusion about the latent structure (categorical vs. dimensional) or reported sufficient information (e.g., CCFIs) to infer a conclusion.

Exclusion criteria were.

1. The article was theoretical, methodological, simulation study without empirical data;
2. The study did not analyze behavioral data;
3. The study was a review and/or meta‐analysis.

The search was limited to published, peer‐reviewed journal articles, as we aimed to provide a critical review of the existing high‐quality literature. Only one potentially relevant non‐journal record was identified and, while excluded from the systematic review, is commented separately in the Discussion.

### Screening and coding

Two independent human coders (MC and RP) screened and coded all articles. All discrepancies were resolved through discussion and were later reviewed by the PI (ET).

Since a single article could report multiple (sub)samples and/or taxometric analyses, the full coded dataset followed a long‐form data structure, with articles appearing across multiple rows. Coded variables were defined in the data dictionary included in the preregistration protocol and served as an ad hoc risk of bias assessment tool. A few fields were added for coder notes and agreement metrics. In addition to taxometric outcomes, we coded whether analyses involved artificial admixture (e.g., case‐control or diagnosis‐structured sampling) as a key risk factor for pseudotaxonic patterns.

Inter‐coder agreement was fair to good across all phases. In the title and abstract screening phase, Cohen's *κ* = 0.79 (34 out of 38 independent decisions matched). In the full‐text eligibility screening, Cohen's *κ* = 0.52 (15 out of 19). For the final data extraction, 84% of rows (71 out of 85) were extracted by both coders. Agreement on critical variables was high: for the “authors' taxonic conclusion” agreement was 94%; for “artificial admixture”, it was 95% at the (sub)sample level.

### Data synthesis

Findings were synthesized descriptively across studies, considering taxometric conclusions together with methodological features relevant to their interpretation. No meta‐analysis was performed because few independent studies were available for each condition, many analyses were dependent within samples, and studies differed substantially in taxometric procedures, indicators, and risk of bias, especially artificial admixture. Although quantitative synthesis of taxometric findings is feasible in larger literatures (Haslam et al., 2020), a pooled estimate was considered difficult to interpret in this small and heterogeneous evidence base.

## RESULTS

Across both searches, 126 records were retrieved (110 from the main database search, 16 from a targeted supplementary search). In the main database search, after removing 67 duplicates and excluding 5 non‐journal records (dissertations or book chapters, only one of which appeared to include primary taxometric analyses, but could not be retrieved), 38 articles were screened for title/abstract, 19 were excluded at title and abstract, and 19 underwent full‐text assessment. Seven were excluded at full‐text for not meeting inclusion criteria. The targeted supplementary search (January 2026) yielded 16 additional records, 2 of which were eligible, resulting in 14 included studies (Figure 1). We proceeded despite the small number of records because the protocol was preregistered and the topic is substantively important.

**FIGURE 1:**
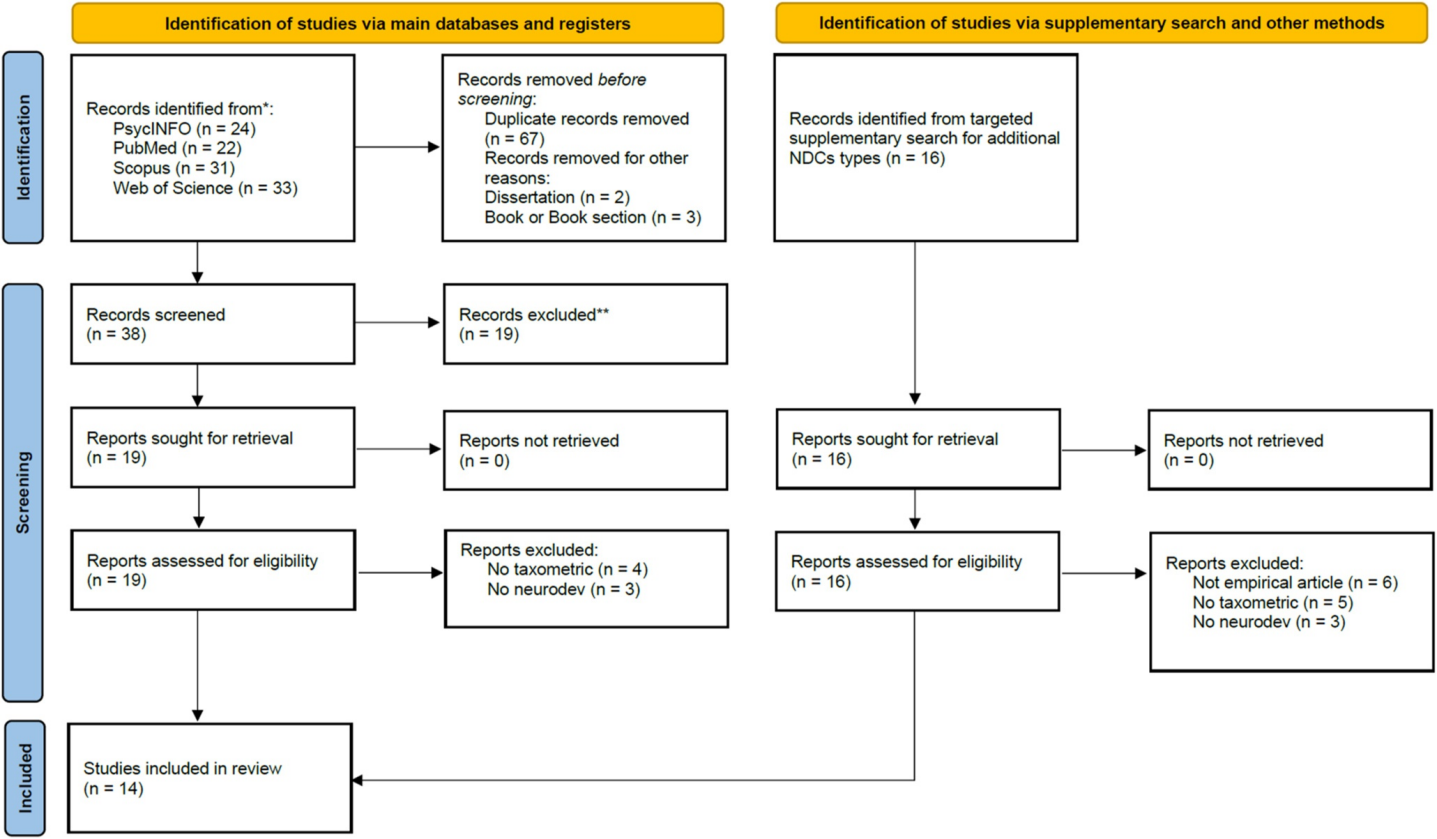
PRISMA 2020 flow diagram, including supplementary identification via online browsing and citation searching.

Fourteen articles were included (15 samples, with multiple analytic subsamples in some studies). Together, they comprised 85 taxometric analyses (range 1–22 per article) and covered ADHD (6 articles), ASD (6, one also examined ADHD), dyslexia (1), and Specific Language Impairment (SLI, 2). Samples ranged from general‐population cohorts to clinically referred and mixed/enriched designs, spanning early childhood to adulthood. Most studies drew on US samples/cohorts, with additional samples from Australia, the United Kingdom, and Qatar. Race or ethnicity was reported in only 4 of 14 studies (29%), where whites/caucasians comprised 56%–88% of the participants. Phenotypes were operationalized as either case status based on clinical/research diagnosis, or as quantitative trait variation; taxometric indicators were typically derived from established rating scales, interviews, as well as cognitive, language, or achievement measures. Ten articles examined disorder‐level taxonicity (i.e., whether a disorder is a discrete taxon in the general population), while four examined putative subtypes (mostly within fully clinically diagnosed samples). A summary of the included studies, simplified to one row per article, is presented in Table 1. Supporting Information S1: Table S1 reports additional study‐level details on sample characteristics, phenotype definition, measurement tools, developmental stage, and ascertainment, while detailed information on all coded taxometric analyses is available in the full Data Extraction Table (archived on OSF: https://osf.io/ys5ad/?view_only=0e2812acfabf43fc8af01f883c7331ba).

**TABLE 1:** Overview summary of the reviewed articles.

Study	Target condition	Taxometric focus	Population analyzed	Country	Age	Taxonic conclusion	Artificial admixture/compound sampling	Sample size	No. of taxometric analyses	Taxometric methods	Interpret. based on	No. of indicators	Indicators skewn.	Nuisance covar.	Indicators validity
Marcus et al. (2012)	ADHD	Disorder	Mixed clinical and non‐clinical	USA^a^	18–65 years	No	Yes	432–600	6	MAMBAC; MAXSLOPE; MAXEIG; L‐Mode	CCFI	2–9	Unreported	Unreported	2.04–3.26
Haslam et al. (2006)	ADHD	Disorder	General population	Australia	6–12/13–17 years	No	No	1222–1774	2	MAXEIG; MAMBAC	Fit_d_	3	0.45–1.49	−0.03–0.23	2.31–3.29
Frazier et al. (2007)	ADHD	Disorder	Clinical referrals	USA	5–66 years	No	No	191–394	22	MAMBAC; MAXEIG; L‐Mode	Fit_d_	3–9	−1.09–0.47	−0.21–0.31	1.03–3.69
Marcus and Barry (2011)	ADHD	Disorder	General population	USA	Children: Grade 3	No	No	667–1078	15	MAMBAC; MAXEIG; L‐Mode	CCFI	2–9	0.74–3.91	−0.12–0.35	1.87–3.26
Stevens et al. (2018)	ADHD	Subtypes	Mixed clinical and non‐clinical	USA^a^	12–18 years	Yes	Partly yes	71–251	2	MAMBAC; MAXSLOPE	CCFI	3	Unreported	Unreported	Unreported
Deserno et al. (2023)	ADHD/ASD	Disorder	Mixed clinical and non‐clinical	USA^a^	7–13 years	No for ADHD, mostly yes for ASD	Likely yes	203–434	4	L‐Mode; MAMBAC; MAXEIG	CCFI	3	Unreported	Unreported	1.86–2.74
Frazier et al. (2023)	ASD	Disorder	Mixed clinical and non‐clinical	Qatar and USA^a^; unreported for other datasets	1.5–22 years	Yes	Yes	512–16755	10	MAMBAC; MAXEIG; L‐Mode	CCFI	3–8	0.19–0.99	0.14–0.73	0.57–1.84
Frazier et al. (2010)	ASD	Disorder	Clinical and unaffected siblings	USA	*M* = 8.17 years (SD = 4.07)	Yes	Partly yes	1825–11472	6	MAMBAC; MAXEIG	CCFI	3–4	Unreported	Unreported	Unreported
James et al. (2016)	ASD	Disorder	General population	UK^a^ (lab; part of online sample)/unreported (social media respondents)	17–47 (lab)/16–70 (online) years	Yes	Yes	1139	1	MAMBAC; MAXCOV; MAXEIG; L‐Mode	CCFI	4	−0.33–0.73	−0.09–0.28	1.29–2.33
Munson et al. (2008)	ASD	Subtypes	Clinical	USA^a^	2.0–5.5 years	Yes	no^b^	456	1	MAXCOV	Visual	4	Unreported	Unreported	Unreported
Ingram et al. (2008)	ASD	Subtypes	Clinical	USA (thompson; inferred for AGRE)	*M* ≈ 8 years	Partly yes	no^b^	481–2254	7	MAMBAC; MAXCOV	CCFI	Unclear	Unreported	Unreported	1.51–2.55
O'Brien et al. (2012)	Dyslexia	Subtypes	Clinical	Unreported	6.5–8.5 years	Mostly yes	Partly yes	488–671	6	MAMBAC; MAXEIG; L‐Mode	CCFI	3–4	Unreported	Unreported	0.82–2.37
Dollaghan (2004)	SLI	Disorder	General population	USA	3 and 4 years	No	No	620–623	2	MAMBAC	Visual	2	Unreported	0.08–0.32	1.93–2.81
Dollaghan (2011)	SLI	Disorder	General population	USA	5.9–6.5 years	No	No	601	1	MAMBAC	Visual	2	Unreported	−0.14 to 0.11	3.47–3.52

*Note*: Methodological variables serve as ad hoc risk of bias assessment.

^a^ Country was inferred from a clearly identified recruitment setting or cohort when participant country was not explicitly stated. Race/ethnicity was reported in only four studies; following the terminology used in the original reports: Marcus et al. (2012), “55.8% Caucasian, 33.7% African‐American, and 10.5% neither Caucasian nor African‐American”; Frazier et al. (2010), “88.1% Caucasian, 11.8% other races, and 0.1% unknown”; Dollaghan (2004), “65% White at age 3% and 66% White at age 4”; and Dollaghan (2011), “68% White, 29% Black, and 3% other/unknown”. Race/ethnicity composition was unreported in the remaining 10 studies.

^b^ Compound sampling cannot in principle be ruled out when targeting subtypes within entirely clinically diagnosed samples, as explained in the Discussion. Additional detailed study characteristics are reported in Supporting Information S1: Table S1.

MAMBAC was the most frequently used procedure, applied in 13 of 14 articles and in 84 of 85 analyses. Other procedures were MAXEIG (9 articles), L‐Mode (7), MAXCOV (3), and MAXSLOPE (2). Most studies employed more than one taxometric procedure, with a median of 3 procedures. Inference relied on fit indices (Fit_d_ or CCFI) in 11 of 14 articles, while the remaining 3 relied on visual inspection of curves. The number of indicators per analysis ranged from 2 to 9 (median was 3, excluding one article where this was unclear). The median sample size across all analyses was 600, or 611.25 when averaged within articles.

At the article level, studies targeting ADHD as a disorder consistently supported dimensionality (5 of 5), as did both studies on language impairment/SLI (2 of 2). In contrast, disorder‐level ASD studies predominantly supported taxonic patterns, but these conclusions were largely based on diagnosis‐structured or mixed/compound samples. The remaining 4 articles were subtype‐focused, and they reported taxonic patterns, either predominantly (Stevens et al., 2018 on ADHD subtypes; Munson et al., 2008 on ASD subtypes; O’Brien et al., 2012 on dyslexia subtypes), or depending on the indicators subset (Ingram et al., 2008 on ASD subtypes). In total, 8 out of 14 articles reached a taxonic conclusion in at least some analyses. At the analysis level, 28 of 85 analyses (32.9%) yielded taxonic results, most often in ASD analyses (21 of 29; 72.4%). Disorder‐level ASD studies reported taxonic findings across publication years until very recently (Deserno et al., 2023; Frazier et al., 2010, 2023; James et al., 2016).

### Risk of bias for taxometric inference

Taxonic conclusions closely followed diagnosis‐structured compound sampling/artificial admixture, while community‐based designs were predominantly associated with dimensional conclusions. Artificial admixture was evident or strongly suspected in 7 of 14 articles (including all those targeting ASD at the disorder level), and in 31 of 85 analyses. At the article level, 6 of the 7 articles with suspected admixture also reported taxonic findings. At the analysis level, taxonic conclusions occurred in 20 of 31 analyses with evident or suspected admixture (64.5%), compared with 8 of 54 analyses where admixture was likely absent (14.8%).

Reporting of key diagnostic statistics for full methodological assessment was often incomplete. Nuisance covariance could be coded in 7 of 14 articles. It was generally low in six (Dollaghan, 2004, 2011; Frazier et al., 2007; Haslam et al., 2006; James et al., 2016; Marcus & Barry, 2011), whereas it exceeded 0.40 for many indicators in Frazier et al. (2023). Indicator skewness was reported in only 5 articles, where skewness coefficients often approached or exceeded |1| for at least one indicator, but with no clear association with taxonic conclusions. By contrast, indicator validity was more consistently reported: it could be coded in 11 of 14 articles and was almost always large or very large (Cohen's d ≫ 1), consistent with best‐practice recommendations for taxometric analysis.

Low putative taxon base rates could also increase the risk of false‐negative dimensional findings. The concern is greatest for SLI, where base rates of 0.04–0.10 in samples of about 600 correspond to fewer than 60 putative cases; Dollaghan (2011) retained a dimensional result after raising this to 0.16–0.17 (about 100 cases). For ADHD, dimensional findings were also obtained in a clinic‐referred sample with about 72% ADHD cases (Frazier et al., 2007), suggesting that low base rates are unlikely to explain the overall convergence of dimensional findings.

### Additional use of cluster analysis

Six of the 14 articles also used clustering methods (LCA or LPA in 5 articles, community detection in 1). In five cases clustering was run alongside taxometrics (Deserno et al., 2023; Frazier et al., 2007, 2023; James et al., 2016; Munson et al., 2008), while in one it was a secondary analysis (Frazier et al., 2010). All six studies targeted ADHD and/or ASD, and clustering results were interpreted as supporting taxonicity, largely converging with the taxometric conclusions. The main exception was Frazier et al. (2007), where taxometrics supported a dimensional ADHD structure while clustering suggested possible subgroups. Risk of artificial admixture was present in all but two studies (Frazier et al., 2007; Munson et al., 2008).

### Monte Carlo simulation: Impact of artificial admixture

Artificial admixture represents a common and powerful threat to validity, but also one that is easily avoidable by design. In our review, it presented a marked risk of bias for studies supporting the taxonic nature of autism. To illustrate the risks associated with artificial admixture, we simulate a dimensional scenario where clinical and nonclinical participants are separately recruited and merged into a single sample.

A single normally distributed latent variable *X* is generated to represent the clinical trait of interest. A set of observed indicators (*x*1, *x*2, *x*3) are then simulated as linear functions of *X* plus normally distributed random noise, yielding *R*2 = 0.77 for each indicator. Individuals above the 99th percentile of *X* are assumed to receive a diagnosis via global clinical assessment. A sample of *N* = 1000 individuals is drawn from the general population. Given the low base rate, only about 10 individuals are expected to naturally receive a diagnosis. To address this, researchers draw a second sample consisting of an additional *n* = 1000, targeting clinically diagnosed cases, and merge it with the original sample. The full simulation code and data are available in the online materials: https://osf.io/ys5ad/?view_only=0e2812acfabf43fc8af01f883c7331ba.

An instance of the simulation illustrating two observed variables *x*_1_ and *x*_2_, is presented in Figure 2. Color is used to distinguish individuals with and without a diagnosis, but even without this aid, two clusters visually emerge. This apparent separation is misleading: no latent categories exist in the data. The appearance of a categorical structure is a consequence of artificial admixture. In this simulated example, indicator validity is high (Cohen's *d* ≈ 2), suggesting strong separation between groups on each variable. Nuisance covariance is partly problematic: among diagnosed individuals, the correlation between indicators is close to zero (*r* ≈ 0.05), but among non‐diagnosed individuals it is substantial (*r* ≈ 0.60).

**FIGURE 2:**
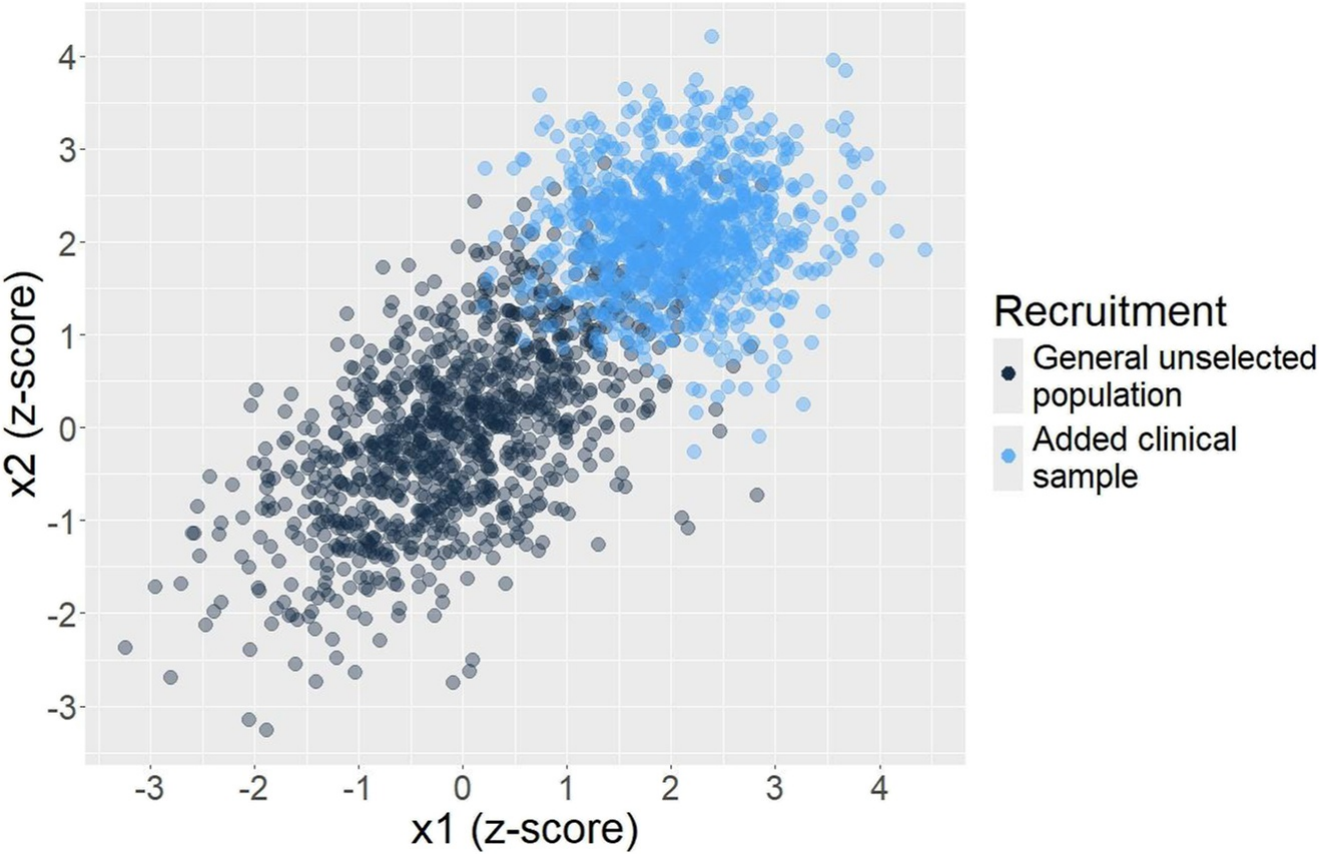
A simulated example of artificial admixture, where a large number of individuals with a diagnosis are separately recruited and added to the sample (total *N* = 2000). The true underlying structure is strictly dimensional.

A Monte Carlo simulation was then conducted with 1000 iterations. Taxometric analysis was performed using the RTaxometric package in R. Inference was based on the CCFI, which ranges from 0 (indicating strong support for a dimensional structure) to 1 (strong support for a categorical structure), with values around 0.5 considered inconclusive. The default procedures (MAMBAC, MAXEIG, and L‐Mode) were applied to each simulated dataset. Three observed indicators were used to enable all procedures.

Over 1000 simulated datasets, results consistently and strongly favored a categorical interpretation: median combined CCFI = 0.88, 95% quantile interval [0.83, 0.92]; median MAMBAC CCFI = 0.90; average MAXEIG CCFI = 0.86; average L‐Mode CCFI = 0.88. The smallest combined CCFI over 1000 iterations was 0.80. Therefore, admixture can be an extremely strong driver of false taxonic findings.

To verify that artificial admixture also produces misleading results when using clustering methods, we ran Gaussian Mixture Models (GMMs) comparing 1‐component versus 2‐component solutions, using the mclust package in R. Model selection was based on the Bayesian Information Criterion (BIC), as per default. GMM is a model‐based clustering method grounded in finite mixture modeling. It is conceptually similar to LPA, but is more flexible as it models covariances among indicators. Across 1000 simulated datasets, results consistently favored the 2‐component solution. The median BIC advantage was 548.67 in favor of the 2‐component model.

To confirm that the categorical structure was incorrectly favored due to artificial admixture, we ran a second Monte Carlo simulation using the same dimensional data‐generating process as before, but without admixture. In this case, a single sample of *N* = 2000 observations was drawn at each iteration. Therefore, nuisance covariance remains present, but there is no admixture. Median combined CCFI = 0.47, 95% quantile interval [0.40, 0.58]; median MAMBAC CCFI = 0.39; average MAXEIG CCFI = 0.49; average L‐Mode CCFI = 0.53. Clustering with GMM consistently favored the 1‐component model (over the 2‐component model), with an average BIC advantage of 30.36 in favor of the 1‐component model.

Thus, without admixture taxometric results leaned towards a dimensional conclusion, but frequently remained inconclusive, probably due to nuisance covariance. It is worth noting, however, that under a dimensional structure achieving both high indicator validity and low within‐group covariance might be practically impossible, because indicators that strongly differentiate individuals with and without a diagnosis must be strongly associated with the underlying trait, thus producing inter‐indicator covariance.

## DISCUSSION

We found that taxometric analysis remains underused in neurodevelopmental research, despite its unique relevance to the ongoing debate about the nature of NDCs (Astle et al., 2022; Happé & Frith, 2021; Michelini et al., 2024; Sonuga‐Barke, 1998, 2020). Across the available literature, only ASD, ADHD, and SLI were targeted as disorders, with no taxometric studies in learning disorders, intellectual disability, or Tourette/tic disorders. Dyslexia was represented by a single study, and only through analyses concerning subtypes. From a clinical standpoint, this gap matters because latent‐structure claims are sometimes used to justify categorical eligibility decisions, yet the evidence base is narrow and methodologically weak.

Taxometric results appeared relatively consistent. Dimensional conclusions were predominantly reached for ADHD and SLI, while categorical conclusions prevailed for ASD and studies examining subtypes of any disorder. This may seem to offer a compelling answer to the debate raised by Chown and Leatherland (2021) and Happé and Frith (2021). However, a closer inspection of the methodological limitations in the reviewed studies suggests that such conclusions should be interpreted with caution.

We identified four disorder‐level ASD studies, all of which reported taxonic conclusions, with some degree of artificial admixture. This represents a major threat to validity of the conclusions of these studies, as demonstrated both in our data simulation and in prior literature (Ruscio & Ruscio, 2004a, 2004b). Admixture in the reviewed studies ranged from sampling participants through different methods, only some of which explicitly targeted clinical populations (James et al., 2016), to combining clinical and non‐clinical samples (Deserno et al., 2023; Frazier et al., 2023). In one case, inclusion criteria from a larger database were partly unclear, but the sample was explicitly structured by diagnostic status (Deserno et al., 2023). Another form of admixture involved constructing a sample of affected and unaffected siblings (Frazier et al., 2010). According to our interpretation, the only relatively unbiased taxometric evidence suggesting that ASD may be a taxon as a disorder comes from a secondary analysis by Frazier et al. (2010), in which only unaffected siblings were examined. As nearly all taxonic claims for ASD derived from structured/compound samples, they provide weak evidence for a natural categorical boundary on phenotypic grounds.

In our simulated example, we showed that artificial admixture represents a major threat to the validity of taxometric analysis, and to clustering methods as well. One might argue that our simulated scenario, in which half of the sample was separately recruited, was extreme. However, this design is broadly consistent with the degree of admixture observed in the reviewed literature.

Changes in autism diagnostic criteria and ascertainment over time may affect sample composition, but a direct comparison of older versus newer definitions could not be made from the reviewed literature: publication year is an imperfect proxy, as recent analyses combined data across periods and frameworks (e.g., Frazier et al., 2023), and James et al. (2016) assessed autistic traits rather than DSM‐defined diagnosis. No reviewed study directly tested whether taxonicity of autism was concentrated among people with intellectual disability, greater impairment, or high‐impact genetic variants. Related within‐ASD analyses identified IQ‐based classes (Munson et al., 2008) and physical‐phenotype classes (Ingram et al., 2008), but these reflect heterogeneity within autism rather than the disorder boundary.

Behavioral‐genetic evidence also supports substantial continuity across the clinical boundary. Robinson et al. (2011) found similar genetic influences across the population distribution and extreme autistic‐trait groups; Colvert et al. (2015) found substantial genetic overlap between continuous autistic traits and clinical ASD measures in a population‐based twin sample. This evidence is compatible with dimensional liability, although etiological continuity does not by itself establish dimensional phenotypic structure.

Concerning subtypes, the situation was similar. All four reviewed articles reached some taxonic conclusions, but two of them likely involved some form of artificial admixture. However, the issue appeared to be nuanced and less severe in this subset of studies. For example, O’Brien et al. (2012) used psychometric cutoffs across different tools and alternate inclusion criteria, which may not necessarily have biased the results, unless the selection criteria correlate with different underlying cognitive impairments in dyslexia (phonological vs. non‐phonological). However, the authors themselves acknowledged this possibility, writing that *“depending on the dyslexia criterion used, samples may vary in composition with regard to their subtypes”* (33). A subtle and potentially overlooked cause of admixture in studies targeting subtypes within entirely clinical samples is that the diagnoses might be based on exceeding cutoff thresholds on partly independent dimensions/scales, reflecting a variant of the second type of problematic sampling listed by Ruscio and Ruscio (2004a) as reported in the introduction. In general, subtype taxonicity should not be conflated with stable, treatment‐relevant groups, and it should not be credited unless it predicts longitudinal outcomes or differential response beyond severity or other known factors.

Low base rates remain a caveat for the limited SLI evidence, although sensitivity analyses in Dollaghan (2011) retained dimensional findings at higher base rates. For ADHD, dimensional results were also obtained in samples with substantially higher base rates. It should be noted that base rate and admixture are often confounded across studies, since admixture is typically used to raise base rates. Overall, the presence of artificial admixture was most frequently associated with taxonic conclusions. Only two notable mismatches were identified: Marcus et al. (2012) reached a dimensional conclusion regarding ADHD despite having incurred admixture of clinical cases, while Munson et al. (2008) reached a taxonic conclusion about ASD subtypes without evidence of admixture (all children had received an ASD diagnosis based on a seemingly uniform clinical assessment). Together, these patterns support a robustly dimensional interpretation for ADHD across designs. For ASD, categorical evidence appears to prevail, but any definitive conclusion is frustrated by sampling‐related biases, especially artificial admixture, and by plausible etiological heterogeneity that taxometrics on behavioral indicators cannot cleanly separate from ascertainment artifacts.

For ADHD and language impairment, where the evidence converges on dimensionality, continuous structure does not preclude diagnostic decisions. Symptom severity, functional impairment, risk, and expected benefit from support can justify practical thresholds for treatment and access to services even when underlying traits are continuous. Such thresholds need not correspond to natural categorical boundaries. ADHD‐related impairment, for example, increases continuously with symptom severity, without evidence of a discrete threshold (Arildskov et al., 2022). Taxonic findings should therefore support claims about natural boundaries only when they provide credible evidence for genuine discontinuity; for autism, the biases reviewed above mean this standard is not yet met.

## CONCLUSIONS AND FUTURE DIRECTIONS

The small number of studies identified, and the virtual lack of studies exploring taxa within learning disorders and other conditions such as intellectual disability, while limiting the potential scope of our review, are themselves informative, highlighting the need for more taxometric investigation in the field. Our exclusion criteria specified the lack of use of behavioral indicators; however, no study was excluded on this basis. One record was screened out for being a dissertation (Clemons, 2006); however, the full document could not be retrieved. Based on its abstract, it focused on whether ADHD subtypes represent distinct taxa, and it reached a taxonic conclusion, but some degree of admixture was possible (as participants were separately recruited from clinical centers and schools). In conclusion, we do not believe that more studies would be included by relaxing eligibility criteria.

The evidence base is also limited in terms of geographical, ethnic, and age coverage. Most studies drew on US samples or other high‐income settings, while race/ethnicity was reported in only four studies. Age coverage was uneven across conditions: ADHD findings were dimensional across child, adolescent, and adult samples, autism showed taxonic findings across studies including both younger and adult participants, whereas language impairment was examined only in children. However, the small number of studies and differences in sampling and study characteristics prevent any clear conclusion about whether taxometric results vary with age. Future studies should include more geographically and demographically diverse samples and examine developmental differences directly across the lifespan.

A relevant future direction concerns a systematic examination of the use and results of cluster analysis, which was employed by some of the reviewed articles, and is a prominent tool in this field (Astle et al., 2022). Model‐based approaches, such as latent class/profile analysis, were the most frequently used. Clustering algorithms are not without problems. First, while they are more flexible and can address broader questions, they do not offer direct or consistent tests of taxonicity (Beauchaine, 2003; Ruscio & Ruscio, 2004b). Second, meaningful scientific inference from clustering (beyond its use as a mere data reduction technique) requires careful handling of assumptions, as the risk of detecting pseudoclusters is high (Toffalini et al., 2022, 2024). Finally, it is not immune to risks such as artificial admixture. Nonetheless, clustering and taxometric analysis could be more directly, and critically, compared when addressing the question of whether neurodevelopmental conditions reflect categories or dimensions.

A crucial implication of our work concerns how taxometric findings should inform traditional diagnostic categories of NDCs such as those in the DSM‐5, which have come under criticism (Astle et al., 2022; Sonuga‐Barke, 2020, 2022). Although described as atheoretical and agnostic about etiology, the DSM operationalizes disorders in ways that reinforce a neo‐Kraepelinian medical paradigm, implicitly reflecting the idea that NDCs reflect discrete categories underpinned by specific dysfunctions. This view continues to shape research and clinical practice (Coghill & Sonuga‐Barke, 2012). Dimensional latent structure is compatible with the practical utility of diagnostic systems, but it requires that researchers and clinicians make their underlying models explicit and do not mistake diagnostic categories for natural taxa. High‐quality taxometric studies can test this assumption directly and deserve a more central role in NDC research.

## AUTHOR CONTRIBUTIONS

**Enrico Toffalini**: Conceptualization; formal analysis; funding acquisition; investigation; methodology; project administration; software; supervision; validation; visualization; writing—original draft preparation; writing—review and editing. **Margherita Calderan**: Data curation; investigation; validation; visualization; writing—review and editing. **Riccardo Pagan**: Data curation; investigation; validation; visualization; writing—review and editing. **Valentina Tobia**: Funding acquisition; project administration; supervision; writing—review and editing. **Edmund Sonuga‐Barke**: Conceptualization; methodology; writing—original draft preparation; writing—review and editing.

## CONFLICT OF INTEREST STATEMENT

The authors declare no conflicts of interest.

## ETHICAL CONSIDERATIONS

This study includes data simulation and a systematic review of previously published research. Therefore, it did not involve the collection of new data from human participants. Accordingly, informed consent or assent (including parental consent) was not required. The review exclusively relied on secondary analysis of published, anonymized data. Ethical approval from a Research Ethics Committee (REC), Institutional Review Board (IRB), or equivalent body was therefore not sought.

## REGISTRATION DETAILS

The review protocol was preregistered on 31 March 2025 in the OSF Registries (registration number: “65Y9G”) and is publicly available at: https://osf.io/65y9g/?view_only=91e97c6b00dc444abb83fefcf67cbac0.

## Supporting information

Table S1

## Data Availability

All data and code that support the findings of this study (including both the systematic review and the data simulation) are openly available in the Open Science Framework (OSF) at http://doi.org/10.17605/OSF.IO/YS5AD, reference number “YS5AD”. Open Data and Code are also publicly available on GitHub at: https://github.com/EnricoToffalini/Taxometrics‐Neurodevelopmental‐Disorders.
