# Supplementary material for: Categories or dimensions? A critical systematic review of taxometric evidence in autism, ADHD, and language disorders
Source: JCPP Adv. 2026 Oct 8:e70177. Online ahead of print. doi: 10.1002/jcv2.70177 (PMC13650027; doi:10.1002/jcv2.70177)
Supplement: Supplementary file 1 [file JCV2-9999-e70177-s001.docx]

# **Categories or Dimensions? A Critical Systematic Review of Taxometric Evidence in Autism, ADHD, and Language Disorders**

**Supporting Information**

**TABLE S1. Expanded Study Characteristics**

| **Study** | **Country** | **Age details** | **Recruitment strategy** | **Exclusion criteria and n excluded** | **Diagnosis details (threshold / impairment criteria)** | **Indicator domain(s)** |
| --- | --- | --- | --- | --- | --- | --- |
| Marcus et al. (2012) | USA (inferred) | Adults; men (n=322) mean 35.3 (SD 9.6), range 18–65; women (n=278) mean 34.2 (SD 10.0), range 18–58; Total: N=600 | Mixed/community+clinic: recruited from clinical settings and newspaper advertisements. Two groups: (1) Axis I/II subjects (n=432) - individuals with psychosocial difficulty related to Axis I and/or Axis II conditions, (2) Healthy Control subjects (n=168) - no current or life history of any Axis I or II disorder. | Physically healthy subjects; other exclusions not reported. n excluded: Not explicitly reported in exclusion criteria section | No formal ADHD diagnosis;  ADHD symptoms measured by self-report Wender-Reimherr Adult Attention Deficit Disorder Scale; impairment criterion not reported | ADHD symptoms: Wender-Reimherr Adult Attention Deficit Disorder Scale (WRAADDS) inattention and hyperactivity/impulsivity indicators |
| Haslam et al. (2006) | Australia | Children 6–12, N=1774 (904 boys, 870 girls); Adolescents 13–17, N=1222 (605 boys, 617 girls); total N=2996 | General population: Participants drawn from National Survey of Mental Health and Well-being of Australia. Participation rate: 86%. | Exclusion criteria: age < 6 years (to ensure school age), missing data on study instruments. Excluded: n = 1513 | No clinician diagnosis reported; ADHD assessed via (1) Child Behaviour Checklist (CBCL) - 11-item Attention Problems scale; (2) Diagnostic Interview Schedule for Children Version IV (DISC-IV) - structured interview administered to parents by trained interviewers, assessing DSM-IV-based ADHD symptoms ; impairment criterion not reported | ADHD symptoms: (1) CBCL Attention Problems total score (parent rating), (2) DISC-IV inattention symptoms count, (3) DISC-IV hyperactivity/impulsivity symptoms count. |
| Frazier et al. (2007) | USA | Children–adults; mean age 14.96 (SD 9.70), range 5–66; Clinical diagnostic groups: Non-ADHD (n = 123, M age 16.67 years, SD 12.13), ADHD Inattentive (n = 86, M age 13.26 years, SD 7.30), ADHD Combined (n = 228, M age 14.69 years, SD 8.89); total N=437 | Clinic-referred: Cleveland Clinic Neuropsychological Assessment Registry (patients referred for neuropsychological evaluation) | Neurological disorders (e.g., seizures, tumors, head injury, infections); FSIQ <70 Excluded n: Not explicitly stated; final sample of 437 represents all evaluations meeting inclusion criteria during 4-year period. N excluded from analyses for specific indicator sets varied | DSM-IV clinical ADHD diagnosis from neuropsychological evaluation + interview; for <18, required cognitive/behavioral symptoms of ADHD in ≥2 settings; impairment criterion for adults not reported | (1) Achievement tests (Woodcock–Johnson Tests of Ability (3rd ed.); (2) Conners Parent ADHD Rating Scale ; (3) Conners Continuous Performance Test (CPT; factor scores and validity indices); (4) Wechsler Intelligence Scales IQ indices and subtests; (5) Neuropsychological processing speed measures; (6) Mixture set (9 highest-validity indicators) |
| Marcus & Barry (2011) | USA | Children, 3rd grade; sample size ranged from 667 to 1078 depending on indicator set and completeness of data | Community-based longitudinal cohort: NICHD Study of Early Child Care and Youth Development (SECCYD) | Missing data excluded (varied by analysis); other exclusions not reported | No clinician diagnosis; ADHD symptoms from parent and teacher reports (CBCL/TRF Attention Problems; DBQ ADHD symptom ratings); impairment criterion not reported | ADHD: CBCL + TRF Attention Problems composites; DBQ parent+teacher symptom ratings; secondary indicators: Classroom Observation System disruptive behavior; Conners CPT omissions+commissions; Tower of Hanoi efficiency; WJ-R Broad Reading+Math average |
| Stevens et al. (2018) | USA (inferred) | Adolescents 12–18 years; mean 15.3 (SD 1.6); N=251 (ADHD n=117; controls n=134) | Community recruitment: recruited via community advertisements; case-control merged (ADHD vs controls) | Medical disorder; drug dependence; current mood/anxiety disorder; lifetime bipolar/OCD/PTSD/Tourette/psychosis/autism/eating disorder; IQ<80; head trauma LOC>1 min; unable to complete 48h medication washout; not safe for MRI; exact n excluded not reported. | DSM-IV ADHD (combined subtype); evaluated with K-SADS-PL; final diagnosis by best-estimate procedure; impairment criterion not reported | Impulsive choice/reward: delay discounting + experiential discounting + SKIP; inhibition/attention/impulsivity tasks: Stop Signal RT; CPT-II commissions; IMT/DMT; Matching Familiar Figures errors |
| Deserno et al. (2023) | USA (inferred) | Children; ADHD mean 9.4 (SD 1.7); ASD 9.3 (SD 1.6); TD 9.4 (SD 1.5); total N=434 | Community-referred cohort: Healthy Brain Network Biobank; diagnosis-structured sampling (ADHD/ASD/TD groups) | Removed missing questionnaire/diagnostic data; removed multivariate outliers (Mahalanobis distance threshold) and 1 univariate outlier | Consensus clinical diagnoses; KSADS-COMP parent+child interviews (DSM-5 based); ADOS used for suspected autism; impairment criterion not reported | ADHD symptoms: SWAN; ASD traits: Social Responsiveness Scale (SRS) |
| Frazier et al. (2023) | Qatar (Autism EYES cohorts: Qatar Foundation) + USA (inferred) (US-Cleveland Clinic); other contributing datasets’ countries not reported | Ages 1.5-22 years; combined datasets (by indicator set): Gaze N=512; ADOS N=8,144–12,705; SRS N=16,755; SCQ N=6,040 | Mixed/enriched secondary datasets aggregated from 7 samples: Autism EYES (Qatar Foundation; US-Cleveland Clinic), SSC, AGRE, NDAR, SRS Norm, Healthy Brain Network; combined ASD and other NDD cases with non-ASD/NDD controls | Exclusion criteria and n excluded not reported (varied by sample) | ASD diagnoses: informant-reported or based on best-estimate clinical/research diagnoses informed by validated gold-standard instruments (Autism Diagnostic Interview-Revised [ADI-R] and/or Autism Diagnostic Observation Schedule [ADOS]). NDD group comprised cases with any neurodevelopmental condition besides ASD. | Social attention (eye-tracking): fixation time %, fixation count, mean fixation duration across 44 stimuli/7 paradigms; ASD symptoms: ADOS (modules 1–4 item-based and sum-based indicator sets), SRS (multiple derived indicator sets), SCQ (lifetime version derived indicators) |
| Frazier et al. (2010) | USA | Total sample mean age 8.17 (SD 4.07); unaffected siblings mean 8.70 (SD 4.28); N=11,507 | Registry/internet: Interactive Autism Network (families with ≥1 child on autism spectrum); siblings design (ASD-affected + unaffected siblings) | Exclusion criteria and n excluded not explicity reported | ASD status per IAN registry (parent report); screening cutoffs noted (SCQ total ≥15; SRS T-score ≥70); impairment criterion not reported | ASD symptoms: SCQ item-group/domain indicators; SRS Social Communication and Autistic Mannerisms indicators |
| James et al. (2016) | United Kingdom (all lab participants and n = 146 online participants) / Not reported (n = 504 Reddit and social-media online responders) | Online sample: range 16–70, mean 26.46 (SD 10.00), N=619; Lab sample: range 17–47, mean 21.42 (SD 4.38), N=523; total N=1142 (N = 1139 analyzed) | Mixed community/student + online: Reddit/social media + University recruitment system | Significant missing data excluded (n=3); otherwise missing data imputed; no mention of IQ, learning/linguistic difficulty or other criteria of exclusions | No formal diagnosis; autistic traits measured by Adult Autism Spectrum Quotient (AQ) self-report; impairment criterion not reported | Autistic traits: AQ-derived composite indicators created via exploratory factor analysis (factor-based subscales) |
| Munson et al. (2008) | USA (inferred from NICHD) | Toddlers/preschoolers 24–66 months; mean 43.4 (SD 8.7); N=456 | Specialty clinic/research program: NICHD Collaborative Programs of Excellence in Autism (multi-site research studies) | Neurological disorder of known etiology; significant sensory/motor impairment; major physical abnormalities; serious head injury n excluded: not explicitly stated | ASD diagnosis using based on: (1) Autism Diagnostic Observation Schedule-Generic (ADOS-G) Module 1 for preverbal children; (2) Autism Diagnostic Interview-Revised (ADI-R) structured parent interview; impairment criterion not reported | Cognition/IQ: Mullen Scales of Early Learning verbal and nonverbal developmental quotients/indices (receptive+expressive language; visual reception+fine motor) |
| Ingram et al. (2008) | USA (Thompson) / unreported (AGRE) | Children with ASD; average age 8 years; Thompson Center sample N=481; AGRE N varied by phenotype (834-2254) | Mixed: registry (AGRE multiplex ASD families) + specialty autism clinic (Thompson Center; consecutive evaluations) | Participants included if they met DSM-IV criteria for ASD and had complete essential/complex classification data. Specific exclusion criteria not explicitly detailed. | ASD; AGRE diagnoses are based on the ADI-R administered by a trained clinical rater; Thompson Center DSM-IV ASD diagnosis (instrument not reported); impairment criterion not reported | ASD symptoms: ADI-R sums (B1-B4; C1+C4) and ADI-R item sets for insistence on sameness + repetitive sensory-motor; language: age first words/phrases; cognition: Raven’s Colored Progressive Matrices + PPVT-III; adaptive: Vineland (VABS); physical: dysmorphology classification + head circumference score |
| O'Brien et al. (2012) | Not reported | Children 6;6–8;6 years; grades 1–3; N=671 | School-based screening/enriched sample: participants in a multi-site reading disability intervention study; identified as having a reading disability via screening measures | No history of primary sensory/neurologic disorders | Diagnostic classifications: Low-achievement criterion (standard score <85 on reading measures); Discrepancy criterion (IQ-achievement discrepancy >1 SD); n excluded not reported. | Phonological processing: (1) CTOPP Elision subtest, (2) CTOPP Nonword Repetition subtest, (3) Sound Symbol Conversion Test, (4) TOWRE Phonemic Decoding subtest. Naming/processing speed: (1) RAN letters (standard score), (2) RAN objects (standard score), (3) WISC-III Coding subtest (scale score) |
| Dollaghan (2004) | USA | Children: age 3 years (N=620, M=37 months, SD=10 months, range 35–42); age 4 years: (N=623, M= 49 months, SD=10 months, range 48–55) | Participants from longitudinal study of otitis media and children's development; age-3 tested subsample drawn from larger cohort | Abnormal hearing on test day n = 82; cognitive SS≤77 (n = 14); missing candidate indicator data (n = 37) | No formal SLI diagnosis; language impairment phenotype examined dimensionally; impairment criterion not reported | Language: (1) PPVT-R (receptive vocabulary - standard score), (2) Mean Length of Utterance (MLU) in morphemes from 15-min conversational language sample, (3) Number of Different Words (NDW) from language sample. expressive vocabulary: (4) Language Development Survey (LDS) total word count by parent report. |
| Dollaghan (2011) | USA | 6-year-olds; M= 72.77 months (SD = 0.85 months), range 71–78; N=601 | General population cohort of healthy singletons from pediatric practices in and near Pittsburgh, Pennsylvania (otitis media longitudinal study) | Failed hearing test (n = 28); WISC FSIQ≤77 (n = 45) or missing (n = 2); missing indicator scores (n = 53); original cohort restricted to normal birthweight/singletons and monolingual English homes | No formal SLI diagnosis; Language deficits defined operationally as scores ≥1.5 SD below mean on language measures; impairment criterion not reported | Language: P1) PPVTR receptive vocabulary (standard score), (2) Mean Length of Utterance (MLU) in morphemes, (3) Number of Different Words (NDW); Phonological memory: (4) Average percentage of phonemes repeated correctly in 3- and 4-syllable nonwords (34PPC) from NRT |
